# Efficacy of Inhaled Treprostinil in a Patient with Systemic Sclerosis-Associated Pulmonary Hypertension and Interstitial Lung Diseases Refractory to Conventional Intravenous Epoprostenol

**DOI:** 10.3390/medicina61020184

**Published:** 2025-01-22

**Authors:** Yuki Hida, Teruhiko Imamura, Ryuichi Ushijima, Koichiro Kinugawa

**Affiliations:** Second Department of Internal Medicine, University of Toyama, 2630 Sugitani, Toyama 930-0194, Japan; time.to.control5@gmail.com (Y.H.);

**Keywords:** heart failure, hemodynamics, congestion

## Abstract

*Background:* Systemic sclerosis-associated pulmonary hypertension (SSc-PH) is widely recognized as the most severe subtype of connective tissue disease-associated pulmonary hypertension (CTD-PH), particularly in patients with complicating factors such as interstitial lung disease (ILD) and biventricular failure. This condition is associated with the poorest clinical outcomes among PH subtypes, presenting significant challenges in both management and prognosis. Despite the use of conventional therapies, including intravenous administration of epoprostenol, a promising prostacyclin analogue, treatment outcomes for SSc-PH remain suboptimal. While epoprostenol has demonstrated efficacy in reducing pulmonary arterial pressures, its clinical application is often constrained by the risk of ventilation–perfusion (V-Q) mismatch, particularly at higher doses. *Case presentation:* We report the case of a 73-year-old woman with SSc-PH complicated by ILD, who experienced progressive hemodynamic deterioration despite receiving optimized therapy with intravenous epoprostenol. Efforts to escalate the dose of epoprostenol were limited by the development of severe V-Q mismatch, precluding further dose increases. In light of these challenges, inhaled treprostinil was introduced as an adjunctive therapy. There were significant improvements in her pulmonary hypertension and hemodynamic parameters, ultimately allowing the discontinuation of intravenous dobutamine and stabilization of her hemodynamics, as well as her respiratory function, exercise capacity, and quality of life. *Conclusions:* This case highlights the potential clinical utility of combining inhaled treprostinil with intravenous epoprostenol for the treatment of SSc-PH in patients with concurrent ILD. By addressing the limitations associated with high-dose intravenous prostacyclin therapy, this combination approach may represent a promising therapeutic strategy for improving outcomes in this difficult-to-treat patient population. Further investigation is warranted to establish the efficacy and feasibility of this combination therapy in larger cohorts of patients with SSc-PH and associated ILD.

## 1. Introduction

Connective tissue diseases (CTDs), including systemic sclerosis (SSc), systemic lupus erythematosus (SLE), and mixed connective tissue disease (MCTD), are frequently associated with pulmonary hypertension (PH) [1]. The pathogenesis of PH in patients with CTDs involves not only inflammatory thickening and sclerosis of the pulmonary arteries but also pulmonary vein stenosis or occlusion, left heart failure, and interstitial lung disease (ILD) [1]. Notably, multiple etiological factors often coexist in patients with SSc. PH associated with SSc (SSc-PH) generally exhibits a worse prognosis compared to idiopathic pulmonary arterial hypertension (IPAH) [2], with outcomes being particularly poor in cases complicated by lung disease [3].

The treatment of SSc-PH typically follows a similar approach to that of IPAH [4]. Continuous intravenous epoprostenol, a prostacyclin analogue, is commonly administered for IPAH, with evidence suggesting that rapid escalation to higher doses yields superior efficacy [5]. However, in SSc-PH, particularly in cases compounded by lung disease, escalating doses of epoprostenol may induce ventilation–perfusion (V-Q) mismatch, thereby restricting the potential for dose optimization. Consequently, the development of an effective and tailored treatment approach for patients with SSc-PH and coexisting lung disease remains an unresolved clinical challenge.

Emerging evidence highlights the potential efficacy of inhaled prostacyclin analogues, though their application in the clinical management of SSc-PH has yet to be comprehensively elucidated [6]. Intravenous prostacyclin is generally indicated in patients with PH assigned to WHO functional class IV. The clinical implication of combination therapy utilizing intravenous/inhaled prostacyclin in this cohort remains uncertain. Here, we present a case study of advanced SSc-PH complicated by ILD, in which a carefully optimized combination of continuous intravenous prostacyclin and inhaled prostacyclin yielded notable clinical benefits.

## 2. Case Presentation

### 2.1. Before Index Hospitalization

The patient was a 73-year-old woman. Thirty-six years ago, she was diagnosed with SSc based on the presence of generalized edematous sclerosis and digital circulatory disturbances, as well as positive antinuclear antibody findings and skin biopsy results. Twelve years ago, she was suspected of having PH due to a tricuspid regurgitation pressure gradient of 45 mmHg. However, ILD was suspected based on chest computed tomography findings as a cause of PH, and its treatment was prioritized. Pulmonary function tests showed a restrictive pattern (%vital capacity 59%) and reduced diffusing capacity for carbon monoxide (%DLCO 23.5%), leading to the initiation of home oxygen therapy at 2.0 L/min.

Nine years ago, she was initially referred to the cardiology department due to resting dyspnea, hypoxemia, and hypotension. She was diagnosed with SSc-PH classified as WHO functional class IV. Right heart catheterization showed mean pulmonary artery pressure 60 mmHg, pulmonary artery wedge pressure 10 mmHg, and pulmonary vascular resistance 18.2 Wood units (WU) (Table 1). Respiratory test showed %vital capacity 49%, forced expiratory volume in one second 80%, and %DLCO 23.5%.

Treatment with intravenous epoprostenol, oral sildenafil, and oral bosentan was initiated, followed by oral nintedanib for concomitant ILD. Arterial–alveolar oxygen tension difference (A-a DO2) was 51 mmHg under 20 ng/min/kg of epoprostenol. The dose of epoprostenol was increased to 38.5 ng/min/kg. One year ago, hypoxia worsened, likely due to V-Q mismatch (A-a DO2 was increased up to 62 mmHg). An increased A-a DO2 declined to 52 mmHg following a reduction in epoprostenol down to 34.8 ng/min/kg. Her symptoms were well managed by a relatively lower dose of epoprostenol.

### 2.2. On Admission

She was urgently admitted to our institute complaining of dyspnea on exertion, bilateral leg edema, and body weight gain. She had received sildenafil 60 mg/day, bosentan 250 mg/day, epoprostenol 26 ng/kg/min, furosemide 60 mg/day, tolvaptan 3.75 mg/day, spironolactone 75 mg/day, and nintedanib 300 mg/day.

Her body height was 158.3 cm, and body weight was 42.2 kg. Blood pressure was 91/60 mmHg, and pulse rate was 85 bpm. Oxygen saturation was 93% under 4.0 L/min of oxygen supply. IIp sound was intensified in auscultation. Fine crackles were diffusely heard in lung auscultation. Bilateral jugular dilatation and leg edema were identified.

Serum albumin was 2.9 mg/dL, hemoglobin was 8.9 g/dL, and estimated glomerular filtration rate was 22.7 mL/min/1.73 m^2^. Serum C-reactive protein was 2.15 mg/dL, and Krebs von den Lugen-6 was 3836 U/mL. Plasma B-type natriuretic peptide was 187 pg/mL.

**Figure 1:**
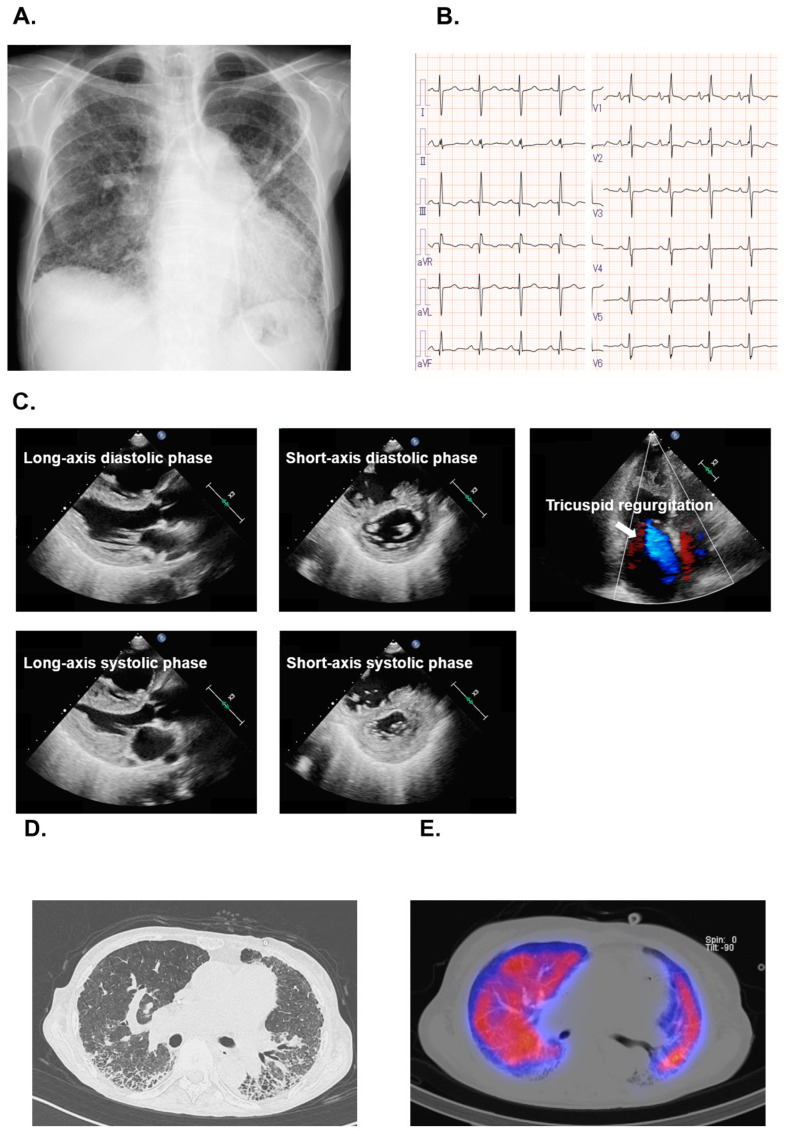
(**A**) Chest X-ray showing cardiomegaly (cardiothoracic ratio of 58%), pulmonary artery dilation, and bilateral reticular and linear opacities. (**B**) Electrocardiogram showing a heart rate of 94 bpm, sinus rhythm, right axis deviation, and an incomplete right bundle branch block. (**C**) Transthoracic echocardiography showing preserved left ventricular systolic function without left ventricular enlargement and elevated left ventricular filling pressure. Left ventricle showing D shape during both diastolic and systolic phase, indicating elevated right ventricular loading. The four-chamber view showing mild to moderate tricuspid regurgitation and enlarged right heart. (**D**) High-resolution chest computed tomography showing bilateral reticular opacities and honeycombing. (**E**) Pulmonary perfusion scintigraphy showing reduced lung perfusion in areas of interstitial shadows.

Chest X-ray and electrocardiogram are displayed in Figure 1A,B. The chest X-ray revealed cardiomegaly (cardiothoracic ratio 58%), pulmonary artery dilation, and bilateral reticular and linear opacities. The electrocardiogram showed a sinus rhythm, heart rate of 94 bpm, right axis deviation, and incomplete right bundle branch block, as well as right heart and bi-atrial hypertrophy. Transthoracic echocardiography showed left ventricular end-diastolic diameter of 37 mm, left ventricular ejection fraction of 52% with a D-shaped left ventricle, tricuspid annular plane systolic excursion of 16 mm, and right ventricular fractional area change of 26% (Figure 1C). The degree of tricuspid regurgitation was mild to moderate with a tricuspid regurgitation pressure gradient of 44 mmHg.

High-resolution chest computed tomography and pulmonary perfusion scintigraphy are displayed in Figure 1D,E. High-resolution chest computed tomography showed bilateral reticular opacities and honeycombing. On pulmonary perfusion scintigraphy, lung perfusion was reduced in the areas of interstitial shadows; however, no evidence of V-Q mismatch was noted. Taking these all together, she was diagnosed with worsening biventricular failure due to advanced SSc-PH and ILD.

### 2.3. In-Hospital Course

**Figure 2:**
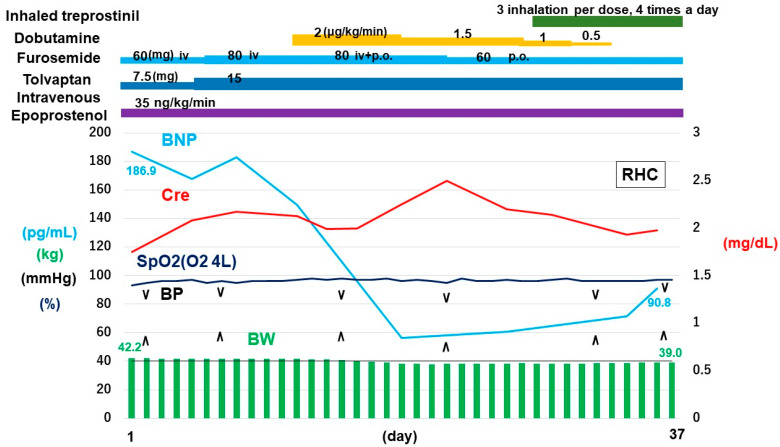
Clinical course. BP: blood pressure; BW: body weight; SpO2: saturation of percutaneous oxygen; Cre: creatinine; BNP: plasma B-type natriuretic peptide; RHC: right heart catheterization; iv: intravenous injection.

We converted oral furosemide to an intravenous one to manage systemic congestion, together with dose up-titration of tolvaptan (Figure 2). We further initiated 2 μg/kg/min of intravenous dobutamine for refractory bi-ventricular failure. Plasma B-type natriuretic peptide level was relatively decreased, whereas desaturation persisted. Epoprostenol could not be up-titrated due to the previous history of V-Q mismatch despite the worsening of SSc-PH. We finally decided to add treprostinil inhalation (72 μg per day) in addition to low-dose epoprostenol.

### 2.4. Treprostinil Inhalation Therapy

The last right heart catheterization finding obtained 2 years ago under epoprostenol 39 ng/kg/min support is displayed in Table 1: mean pulmonary artery pressure 52 mmHg, pulmonary artery wedge pressure 14 mmHg, and pulmonary vascular resistance 9.25 WU.

Following the initiation of treprostinil inhalation together with epoprostenol 35 ng/kg/min, mean pulmonary artery pressure was 43 mmHg, pulmonary artery wedge pressure was 9 mmHg, and pulmonary vascular resistance was 7.85 WU. Desaturation was not encountered, indicating no worsening of V-Q mismatch following the initiation of treprostinil inhalation. Intravenous furosemide was returned to the oral one, and intravenous dobutamine was weaned off without hemodynamic deterioration.

She was discharged on foot on day 37. After the index discharge, she was followed-up at our out-patient clinic for 12 months without worsening of dyspnea.

## 3. Discussion

In the current case report, we presented a patient with advanced SSc-PH complicated by ILD, in which a carefully optimized combination of continuous intravenous prostacyclin epoprostenol and inhaled prostacyclin treprostinil yielded notable clinical benefits and hemodynamic stabilization without worsening V-Q mismatch.

PH is classified into five groups based on etiology and pathophysiology [4]. PAH associated with CTD is relatively common among secondary causes of PAH, with SSc accounting for approximately 19% of CTD-PAH cases [7]. Notably, SSc-PH is associated with a significantly poorer prognosis compared to IPAH [2] and is considered the most severe form of CTD-related PH. In patients with SSc, multiple factors—including pulmonary arterial degeneration, pulmonary vein stenosis or occlusion, left ventricular diastolic dysfunction, secondary right ventricular failure, and ILD—often coexist, complicating the clinical course and making treatment strategies particularly challenging [1].

The therapeutic approach for SSc-PH follows similar principles to that of IPAH [4], with combination therapy involving endothelin receptor antagonists and phosphodiesterase type 5 (PDE5) inhibitors being recommended, although optimal therapeutic strategy remains unestablished. Prostacyclin therapy is generally added for patients at high risk. High-dose intravenous epoprostenol has demonstrated efficacy in treating IPAH [5]. However, in the context of SSc-PH, aggressive pulmonary vasodilation with high-dose epoprostenol poses the risk of deteriorating oxygenation due to pulmonary congestion or V-Q mismatch, particularly in patients with severe SSC-PH accompanying ILD and heart failure.

Patients with SSc-PH and concomitant ILD face an even worse prognosis compared to those with SSc-PH alone [3]. While various pharmacological treatments have been explored for PH associated with ILD, few drugs have proven to be effective. For instance, sildenafil, a PDE5 inhibitor, failed to improve mortality but showed beneficial effects on secondary outcomes, including arterial oxygenation, diffusing capacity of the lung for carbon monoxide, dyspnea severity, and quality of life in this cohort [8].

More recently, inhaled treprostinil, a prostacyclin analogue, has improved exercise capacity, reduced NT-proBNP levels, and lowered the risk of worsening underlying lung disease [6]. Long-term use of inhaled treprostinil has also proven to be both safe and effective [9]. In contrast to intravenous therapies, inhaled prostacyclin analogues are expected to selectively target ventilated lung regions, thus minimizing the risk of V-Q mismatch. Furthermore, inhalation agents are less invasive than intravenous agents, increasing adherence and decreasing the risk of complications.

Intravenous prostacyclin is generally indicated in patients with WHO functional class IV PH. In cases where escalating prostacyclin doses, such as intravenous epoprostenol, is limited by worsening oxygenation, the addition of inhaled prostacyclin may theoretically help reduce pulmonary vascular resistance without compromising oxygenation, although such a therapeutic strategy has not yet been validated.

In our case, the inhaled treprostinil, in addition to multidisciplinary therapeutic interventions, enabled successful weaning from dobutamine without recurrence of bi-ventricular failure or exacerbation of PH. Recurrence of V-Q mismatch was not encountered following the initiation of inhaled treprostinil in addition to low-dose epoprostenol. Higher doses of inhaled treprostinil have been shown to prevent clinical deterioration and improve outcomes in ILD-associated PH [10]. In certain cases, dose escalation of treprostinil may even allow for a reduction in the intravenous epoprostenol dosage. The findings from this case underscore the need for further research into individualized therapeutic strategies for patients with SSc-PH and concomitant ILD to generalize our findings. Future studies should focus on the long-term efficacy and feasibility of combination therapies involving inhaled prostacyclin analogues and intravenous prostacyclin in larger patient cohorts. Notably, the optimal rate between inhalant and intravenous prostacyclin remains uncertain. Additionally, the role of advanced imaging and biomarkers in guiding the titration of prostacyclin therapies warrants exploration to optimize treatment outcomes while minimizing adverse effects such as V-Q mismatch.

## 4. Conclusions

In a patient with SSc-PH complicated by ILD and a history of worsening V-Q mismatch following high-dose continuous intravenous prostacyclin treatment, who received the addition of inhaled prostacyclin analogue therapy to low-dose continuous intravenous prostacyclin treatment, there was a significant improvement in PH without inducing oxygenation impairment or hypotension. This case underscores the potential of a tailored combination therapy approach to address the complex and multifactorial pathophysiology of SSc-PH with ILD, which is often associated with poor prognoses and limited therapeutic options.

Future research is essential to validate these findings in larger cohorts and to explore the long-term efficacy and safety of this combination strategy. Investigations into personalized treatment approaches, integrating advanced imaging, biomarkers, and molecular profiling, may further refine therapeutic protocols, such as a rate between inhalant and intravenous prostacyclin, for patients with SSc-PH and concomitant ILD.

## Figures and Tables

**Table 1:** Trajectory of the dose of epoprostenol and hemodynamics parameters.

	Epoprostenol (ng/kg/min)	SvO2 (%)	mean PAP (mmHg)	RAP (mmHg)	PAWP (mmHg)	CO (L/min)	PVR (Wood Unit)
Aug., Year X-9	-	46.7	60	16	10	2.75	18.2
Aug., Year X-9	4	64.5	42	7	9	3.3	10
Oct., Year X-9	15	75.6	42	7	10	4.48	7.14
Year X-8	20	77	37	4	10	5.54	4.87
Year X-7	35	74.3	35	9	12	6.7	3.43
Year X-2	39	66.9	52	10	14	4.11	9.25

SvO_2_, mixed venous oxygen saturation; PAP, pulmonary artery pressure; RAP, right atrium pressure; PAWP, pulmonary artery wedge pressure; CO, cardiac output; PVR, pulmonary vascular resistance.

## Data Availability

Data are available upon reasonable request from the corresponding author.
